# A comparative wordlist for investigating distant relations among languages in Lowland South America

**DOI:** 10.1038/s41597-024-02928-7

**Published:** 2024-01-18

**Authors:** Frederic Blum, Carlos Barrientos, Roberto Zariquiey, Johann-Mattis List

**Affiliations:** 1 Department of Linguistic and Cultural Evolution, Max Planck Institute for Evolutionary Anthropology, Leipzig, Germany; 2 University of Leipzig, Leipzig, Germany; 3 Pontificia Universidad Católica del Perú, Lima, Perú; 4 Chair of Multilingual Computational Linguistics, University of Passau, Passau, Germany

**Keywords:** Social anthropology, Interdisciplinary studies

## Abstract

The history of the language families in Lowland South America remains an understudied area of historical linguistics. Panoan and Tacanan, two language families from this area, have frequently been proposed to descend from the same ancestor. Despite ample evidence in favor of this hypothesis, not all scholars accept it as proven beyond doubt. We compiled a new lexical questionnaire with 501 basic concepts to investigate the genetic relation between Panoan and Tacanan languages. The dataset includes data from twelve Panoan, five Tacanan, and four other languages which have previously been suggested to be related to Pano-Tacanan. Through the transparent annotation of grammatical morphemes and partial cognates, our dataset provides the basis for testing language relationships both qualitatively and quantitatively. The data is not only relevant for the investigation of the ancestry of Panoan and Tacanan languages. Reflecting the state of the art in computer-assisted approaches for historical language comparison, it can serve as a role model for linguistic studies in other areas of the world.

## Background & Summary

Much of the human history in South America is unknown, and linguistics can be one of many tools to investigate the human past. Yet, the linguistic history in South America is poorly understood, and despite the comparably recent human settlement, many genetic relationships between language families remain hypotheses without too much evidence^1–3^. In addition to that, most languages on the continent are severely endangered and the linguistic window to human history is closing^4,5^. New possibilities arise through the growth and application of computational methods, which in recent years not only inspired new research questions, but also offer a new perspective on unanswered cases. Part of this perspective has been made possible through transparent annotation of data and larger datasets becoming available^5–10^. Computational methods have become a valuable contribution to the study of linguistic history^11–13^. By combining those methods with the detailed work in documentary and historical linguistics, we aim to re-evaluate long-distance genetic relationships which have been proposed in the 20th century by applying the newly arisen methodologies.

One such case is the hypothesized Pano-Tacanan language family. Panoan and Tacanan are two language families currently spoken in Lowland South America^14,15^, which have long been hypothesized to be genetically related^16,17^. Both language families have also been claimed to be related to other languages in the area, such as Mosetén^18,19^, Chipaya, and Movima^20^. Even though there is a considerable amount of evidence in favor of the ‘Pano-Tacanan hypothesis’^21–23^, no fully accepted large-scale reconstruction has yet been carried out. The Panoan language family was first proposed by de la Grasserie in 1889^24^. A preliminary reconstruction of the common ancestor was carried out by Shell^25^, which, however, lacked data from the Northern branch of the family and of Kaxararí^26^. Recently, a new reconstruction has been proposed by Oliveira^27^, which still needs further revisions. The Tacanan languages on the other hand were proposed by Brinton in 1891^28^ and reconstructed by Key^29^ and later Girard^17^. Based on this reconstruction and the ‘Reconstructed Pano’ from Shell, Girard also proposed a reconstruction for the ancestral language, Proto-Pano-Tacanan. Given the problems of the sampled languages for Shell’s Panoan reconstruction, however, this reconstruction is not generally accepted as a proof for the Pano-Tacanan family, and some doubts remain. More recently, Valenzuela & Zariquiey^23^ provide a new reconstruction of Proto-Pano-Tacanan, but this work is limited with respect to the amount of lexical coverage. It does, however, provide a first detailed account of grammatical morphemes that appear to be cognates between the Panoan and the Tacanan language family. Cognates are lexical roots and morphemes from two genetically related languages that descend from the same ancestral form etymologically^30^.

This dataset aims to present lexical data that can be used as a new starting point for investigating the past of Panoan, Tacanan, and other languages. Using state-of-the-art methods for computer-assisted historical language comparison^13^, our dataset presents lexical data for 501 concepts of basic vocabulary across 21 languages. Basic vocabulary refers to stable lexical terms that are assumed to be more resistant to lexical borrowing than others, and thus more reliable than other parts of the vocabulary for establishing sound correspondences. Together with grammatical evidence, they are generally accepted as providing evidence for genetic relationships between languages^31^.

Of the 21 languages, 17 are directly part of either Panoan or Tacanan, and four languages are included that have previously been claimed to be related to Pano-Tacanan. In total, the dataset comprises data from five different genealogical entities: Panoan, Tacanan, Chipaya, Mosetén-Tsimane, and Movima. The data is annotated for morphemes and partial cognacy, which opens the path for a detailed computer-assisted analysis of the languages involved, both from a qualitative and a quantitative perspective. Examples for such a workflow are provided as Technical Validation and Usage Notes. The dataset is intended to work as a role model for future studies on other long-distance genetic relationships, which can orient themselves at the standards and details of annotation offered in this dataset.

## Methods

### Wordlists for Historical Language Comparison

The original goal for developing the dataset was an analysis of the genetic relationship of the languages involved. In order to conclusively establish such a relationship, a detailed phonological and morphological reconstruction that extends to the grammatical structure of the languages is necessary. However, the long way of proving the genetic relationship between languages tends to start with a comparative wordlist of basic vocabulary that is not specific to any culture or geographic region^32,33^. While the traditional wordlists mostly include 100 or 200 concepts^34,35^, the low lexical coverage has been criticized for several reasons. For purposes of language documentation, Dockum and Bowern^36^ argue that an average of 400 lexical items is necessary to identify all phonemes of a language. This, of course, is also a pre-requisite for an accurate historical analysis. Automated methods have been shown to benefit from wordlists of at least 300 items^37^. Other scholars argue that a minimum number of 500 etymological concepts is necessary in order to find sufficient recurring sound correspondence patterns to work on the phonological reconstruction using the comparative method^2^. For phylogenetic studies, a sample size of 33 cognate classes per classified language has been suggested^38^. In any case, this means that in order to capture all relevant sound correspondences for Pano-Tacanan (and beyond), the first necessary (but not sufficient) step is to create a large-scale lexical dataset.

In its current version, the concept list of our dataset contains data for 501 concepts. The list is largely based on a rarely used concept list proposed by Kaufman (https://www.ailla.utexas.org/islandora/object/ailla%3A246899) of more than 1000 individual entries. This list was originally gathered by analyzing 35 comparative studies that involve reconstruction of ancestor languages. The most frequent of the 2100 meanings compiled from those studies have been selected by the original author for his final list. The author claims that those are the most stable etymologies, and are part of a ‘universal basic vocabulary’ that, ‘if applied to a set of related languages, will yield more true cognates than any other list of its size’ (Kaufman 1973, p.29)^39^. As the original list was only recently made available in an archive, it has not found wide distribution among scholars earlier, despite its potential for historical language comparison. The approach presented by Kaufman contrasts with other approaches to historical language comparison, where the dictionaries are searched in a targeted way for specific items that are assumed to be cognate. However, this way of investigating cognacy among putatively related languages comes at the danger of cherry-picking the desired data. The advantage of the larger wordlist is that it is more realistic to find all the relevant sound correspondences compared to the small wordlists, while not cherry-picking the data.

For creating the first version of our wordlist, we chose a 450-concept subset of the concepts provided by Kaufman. For example, we have removed concepts relating to grammatical concepts (e.g. verbal inflection markers and case-marking) and those that relate for flora and fauna. As one step during the pre-processing of the data, we added the concept list to Concepticon^40^ (https://concepticon.clld.org/contributions/Kaufman-1973-1028). This makes it possible to compare our concept list with concept lists that had been used in previous attempts of reconstructing Panoan (Shell, Oliveira) and Tacanan (Girard) in order to arrive at the most important concepts for this study. For this purpose, the concepts reconstructed for Proto-Panoan^27^ and for Proto-Tacanan^17^ were also added to Concepticon. The addition to Concepticon helps in mapping the individual concepts despite the different languages of the original publications (English, Spanish, Portuguese) and to easily integrate comparisons to other sources, such as the Swadesh lists for basic vocabulary^34,35^. In the selection of the final subset, we have oriented ourselves at the intersection of concepts between the Kaufman-conceptlist and the conceptlists from Swadesh, Oliveira, and Girard. The concepts from Oliveira and Girard that have not been used are primarily terms for flora and fauna, while the more basic terms have been preserved.

### Language Sample

**Fig. 1:**
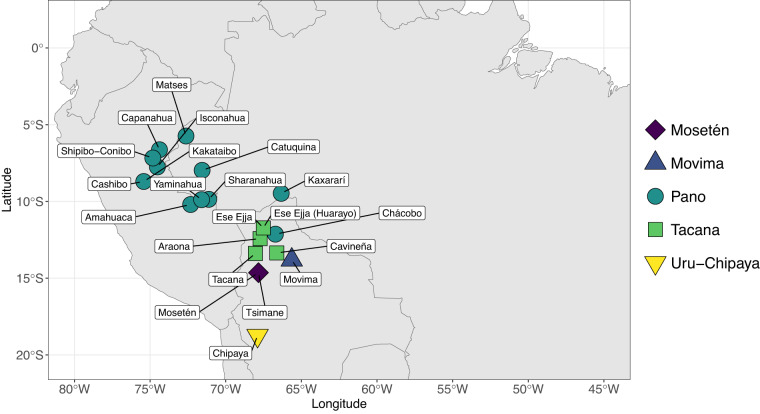
Location of sampled languages in the dataset.

The language sample includes three groups of languages, namely a) twelve (of 18 extant) Panoan, b) five (of seven extant) Tacanan languages, and c) four languages from three small language families, which have previously been argued to be related to Panoan or Pano-Tacanan. All relevant languages for which reliable linguistic material exists have been included in the sample. Given the large amount of concepts in this dataset and the need for high mutual coverage, only languages with published dictionaries have been selected. A more detailed account of the sources will be presented in Table 2 in the Data Records section. The map of sampled languages is presented in Fig. 1.

The data was gathered using both traditional and computational methods. The most important single source is the IDS dataset^41^. This digital publication provided the data for four Panoan (Cashibo, Catuquina, Shipibo-Conibo, Yaminawa) and two Tacanan (Ese Ejja, Huarayo) varieties. As there is a considerable overlap between the IDS conceptlist and ours, it was possible to quickly integrate the data. The availability from IDS also contributed to the integration of non-Panoan languages, as Movima and Mosetén were already digitally available. To complement this, the extensively documented Tsimané variety of Mosetén was added manually to complement the sample. For some languages, we were able to extract the data directly from digital published dictionaries. In two cases (Isconahua, Kakataibo), data recorded by one of the co-authors (RZ) and stored using ToolboX, a language documentation software, were parsed and manually checked for integration into the wordlist. Two other dictionaries, Matses^26^ and Sharanahua^42^, were parsed from their PDF source. For the remaining languages, we went through the dictionaries manually to extract the relevant data.

A recurring problem for the manual sampling is synonymy. There are many cases where the authors give more than two forms for a meaning, which results in a problematically high synonymy. Where possible, we included only the most frequently used item for a specific concept. Such information is often provided in dictionaries, where archaic terms or less commonly used terms are provided after the most frequently used one. In cases of allomorphic or phonological alternations, we have added this as notes to the data entry. In other cases, the most general form has been used, if such information was available. For example, the entry for ‘to eat’ might be accompanied by terms for ‘to eat fish’ or ‘to eat fruit’. In this case, we have chosen the first, most general entry, that provides the best fit for our target concept.

There is one exception to the goal of high coverage, and that is the sparsely documented language Kaxararí. Due to several phonological characteristics absent in other Panoan languages, like the presence of a lateral consonant /l/, Kaxararí is argued to be of great importance for the reconstruction of Proto-Panoan^27^. For this reason, we extracted the data for Kaxararí from another dataset which digitized the Proto-Pano reconstruction by Oliveira^27,43^. In his work, Oliveira includes data on Kaxararí from several different sources, presenting 171 forms in total. Due to the lack of a detailed grammatical description of the language, the exact quality of the available data for this language cannot be confirmed without further documentation work with the speakers. Excluding Kaxararí, the total coverage is at ∼85%.

### Annotation in EDICTOR: Morphemes, Cognates, Alignments

The data is annotated with respect to different levels of linguistic analysis using computer-assisted methodology. For a detailed historic analysis, we need to detect and segment morphemes, and assign partial cognacy to all elements^44^. Furthermore, we need to exclude known borrowings from the data. As only words that descend from the proto-language should be considered cognate, all known borrowings from Spanish and Quechua that could be found in the data were annotated as such.

As a starting point for annotating the data, automated cognate judgements are carried out using the LexStat algorithm from LingPy (v2.6.9^45^). For carrying out the manual annotations and to correct the automated cognate judgements, the data is imported to EDICTOR (v2.0.0)^46^, a visual tool for annotating data in historical linguistics. In a first step, affixes are separated from their roots. They are assigned a different ID of cognacy, as they do not relate etymologically to the lexical root. As part of this step, we have included morpheme glosses^47,48^. In this step, non-salient morphemes are tagged explicitly in order to be excluded from further analysis^44^. This includes for example verbal derivational markers or instrumental nominalizers whose presence is mostly due to artifacts in the process of language documentation. For example, in some traditions verbs are always presented in the first-person singular form, while others may give a base form. Excluding such kind of artifacts from the data is thus essential to assure comparability across forms. After the segmentation of morphemes, partial cognacy is analyzed within each concept^44^. In a second step, this analysis is carried out across similar meanings. For example, many of the languages in the dataset colexify the terms for green and unripe. Hence, the forms are represented in the same cognate set. Other examples include body-part roots, which are widespread across the Panoan languages^49,50^. This is showcased in Table 1, where several languages share the same body-part root for small, round objects (e.g. ‘eye’, ‘seed’), but the formatives differ with respect to the affix they combine with to arrive at different concepts. A network visualization of full colexifications will be presented as part of the Technical Validation.

**Table 1:** Segmented and annotated morphemes various concepts related to eye in Panoan languages.

Concept	Doculect	Segments	Partial cognacy	Morphemes
eye	Amahuaca	β ɨ + r o	681 608	round_object + eye
eye	Capanahua	β ɨ + r o	681 608	round_object + eye
eyebrow	Cashibo	β ɨ + s k o	681 860	round_object + eyebrow
eyebrow	Chacobo	β ɨ + s k o	681 860	round_object + eyebrow
tear (of eye)	Isconahua	β ɨ + r o + ɨ n ɨ	681 608 315	round_object + eye + water

In the final step, all cognate sets that have been found are aligned phonetically. During this step, all cognate sets are checked for validity, and erroneous cognate judgements have been fixed. The correspondence patterns can now be extracted and analyzed computationally as well as in manual fashion. An example for this automated extraction is added as a script within the data repository and briefly presented in the Data Records.

## Data Records

The dataset in its current version is stored on Zenodo (v0.2)^51^. It is published under a CC-BY 4.0 license and curated on GitHub (https://github.com/pano-tacanan-history/blumpanotacana/tree/v0.2). The data follows a specific template of CLDF^7^, namely that of Lexibank^8^. The main data intended for re-use is stored in the ‘cldf’-folder, while the two additional folders ‘raw’ and ‘etc’ are mainly used for the conversion of the raw data into CLDF. In the main directory, the ‘lexibank_blumpanotacana.py’-script manages the conversion from raw data to CLDF. This includes a download of the most up-to-date version of the data from EDICTOR. A ‘metadata.json’ file stores all the relevant metadata for the dataset, namely its ID, a short description, the adequate citation, the license, and the link to the Concepticon wordlist. Further technical details of CLDF will be described in the section on Technical Validation.

The ‘cldf’-folder consists of csvw-files (‘csv on the web’) whose metadata is stored in the ‘cldf-metadata.json’ file. The individual lexemes are stored in ‘forms.csv’, with columns for the entry ID, the language ID, a parameter ID, value and form of the entry, tokenized segments, additional comments that have been added during analysis, the source, the cognate set ID’s, and information about borrowing. The ‘cognate.csv’ file stores additional information about the cognate sets, such as the detection method (‘expert’, because it has been done manually by the first author) and the phonetic alignments of the tokenized entries. A ‘languages.csv’ file includes the necessary information about the languages in the dataset: ID, Name, Glottocode, the Macroarea, Latitude and Longitude of the language, and the language subgroup. Similarly, the ‘parameters.csv’ file stores information about the concepts, their ID, Name, their ID and glosses on Concepticon, as well as a translation to Spanish and Portuguese, common languages for dictionaries which have been used as source for the dataset. This file provides the translational equivalents to the English concepts. The ‘sources.bib’ file contains all the sources that contributed to the dataset in BiBTeX-format. The ‘requirements.txt’ and ‘README.md’ files round off the folder for reproducibility of the CLDF conversion.

The original raw data is represented in a csv-file within the ‘raw’-folder. A metadata ‘etc’ folder includes the tsv-files that are necessary for linking the data to other large-scale linguistic datasets. This includes the mapping of the languages to Glottolog (‘languages.tsv’)^52^ and orthography profiles that map the graphemes in all languages to sounds in CLTS^53^. Those are included within a subfolder that contains the individual orthography profiles for all languages. We have included a folder ‘analysis/‘ which includes all scripts as presented in the Usage Notes. This includes the automated extraction of correspondence patterns (‘s_patterns.py’) using the LingRex package in Python^54,55^, as well the code for all figures that are part of this data descriptor. The main README.md file containts a walk-through for all scripts. The coverage, synonymy, and sources of all languages are presented in Table 2.

**Table 2:** Source, synonymy, and coverage of all language varieties in the dataset.

Variety	Glottocode	Language Family	Original Author	Coverage	Synonymy
Amahuaca	amah1246	Pano	Hyde^63^	0.85	1.15
Capanahua	capa1241	Pano	Loos & Loos^64^	0.91	1.36
Cashibo	cash1251	Pano	Key & Comrie^41^	0.79	1.14
Catuquina	pano1254	Pano	Key & Comrie^41^	0.91	1.06
Chácobo	chac1251	Pano	Zingg^65^	0.93	1.05
Isconahua	isco1239	Pano	Zariquiey^66^	0.77	1.13
Kakataibo	cash1251	Pano	Zariquiey^67^	0.88	1.27
Kaxararí	kaxa1239	Pano	Oliveira^27^	0.21	1.07
Matses	mats1244	Pano	Fleck *et al*.^26^	0.86	1.28
Sharanahua	shar1245	Pano	Scott^42^	0.93	1.30
Shipibo-Conibo	ship1254	Pano	Key & Comrie^41^	0.85	1.38
Yaminahua	yami1256	Pano	Key & Comrie^41^	0.89	1.17
Araona	arao1248	Tacana	de Pitman^68^	0.82	1.03
Cavineña	cavi1250	Tacana	Guillaume^69^	0.74	1.20
Ese Ejja	esee1248	Tacana	Key & Comrie^41^	0.81	1.36
Ese Ejja (Huarayo)	esee1248	Tacana	Key & Comrie^41^	0.74	1.07
Tacana	taca1256	Tacana	Ottaviano & Ottaviano^70^	0.86	1.07
Chipaya	chip1262	Uru-Chipaya	Cerrón-Palomino & Ballón Aguirre^71^	0.87	1.29
Movima	movi1243	isolate	Key & Comrie^41^	0.90	1.00
Mosetén	mose1249	Mosetén-Tsimane	Key & Comrie^41^	0.87	1.14
Tsimane	mose1249	Mosetén-Tsimane	Gill^72^	0.87	1.20
**Overall: 21**				**0.82**	**1.17**

## Technical Validation

### Integration with Reference Catalogues

The final data is presented using the Cross-Linguistic Data Format (CLDF)^7^. The conversion into CLDF includes several control measures, such as the linking to several linguistic reference catalogues to retrieve information about the concepts (Concepticon v3.1.0)^40,56^, the languages (Glottolog v4.8)^52^, and the phonemes in the data (CLTS v2.2.0)^53,57^. This includes the mapping of graphemes to tokenized phonemes through orthography profiles^58^, ensuring that all representations for analysis are based on sounds, and not on orthography. The phonemes are linked directly to CLTS, which contains further information about the individual sounds. Similarly, all concepts in the data are linked to the list on Concepticon^40^. A metadata file for languages includes information such as the glottocode for linking to Glottolog, and information on the language family subgroup. In the two cases of overlapping Glottocodes (Mosetén-Chimané, EseEjja-Huarayo), the two ID’s in the dataset include two recognized varieties that are not represented as such in Glottolog.

### Quality Measures

CLDF comes with a variety of quality measures. The data is converted using cldfbench with the pylexibank plug-in^59^. This step involves detailed quality checks, such as whether all sounds are represented according to the CLTS standard, that all concepts are represented in the conceptlist, and that all languages are part of the languages-metadata file.

**Fig. 2:**
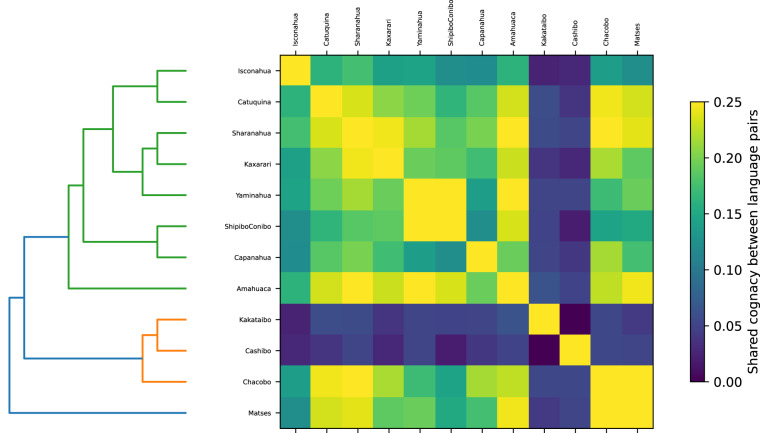
Proportion of shared cognate sets between Panoan languages.

The standardization of the data makes it easy to conduct further computational measures that assure the quality of the data. Based on a computational implementation that measures the regularity of correspondence patterns in cognate sets in the data^60^, we can analyze the proposed cognate sets. In Fig. 2, we present the proportion of shared cognate sets between Panoan languages. Even though this measure is not an explicit phylogenetic representation, it closely resembles the currently accepted family tree for the Panoan languages, with Matses forming an outgroup^26^.

### Visualizing Colexifications in the Data

**Fig. 3:**
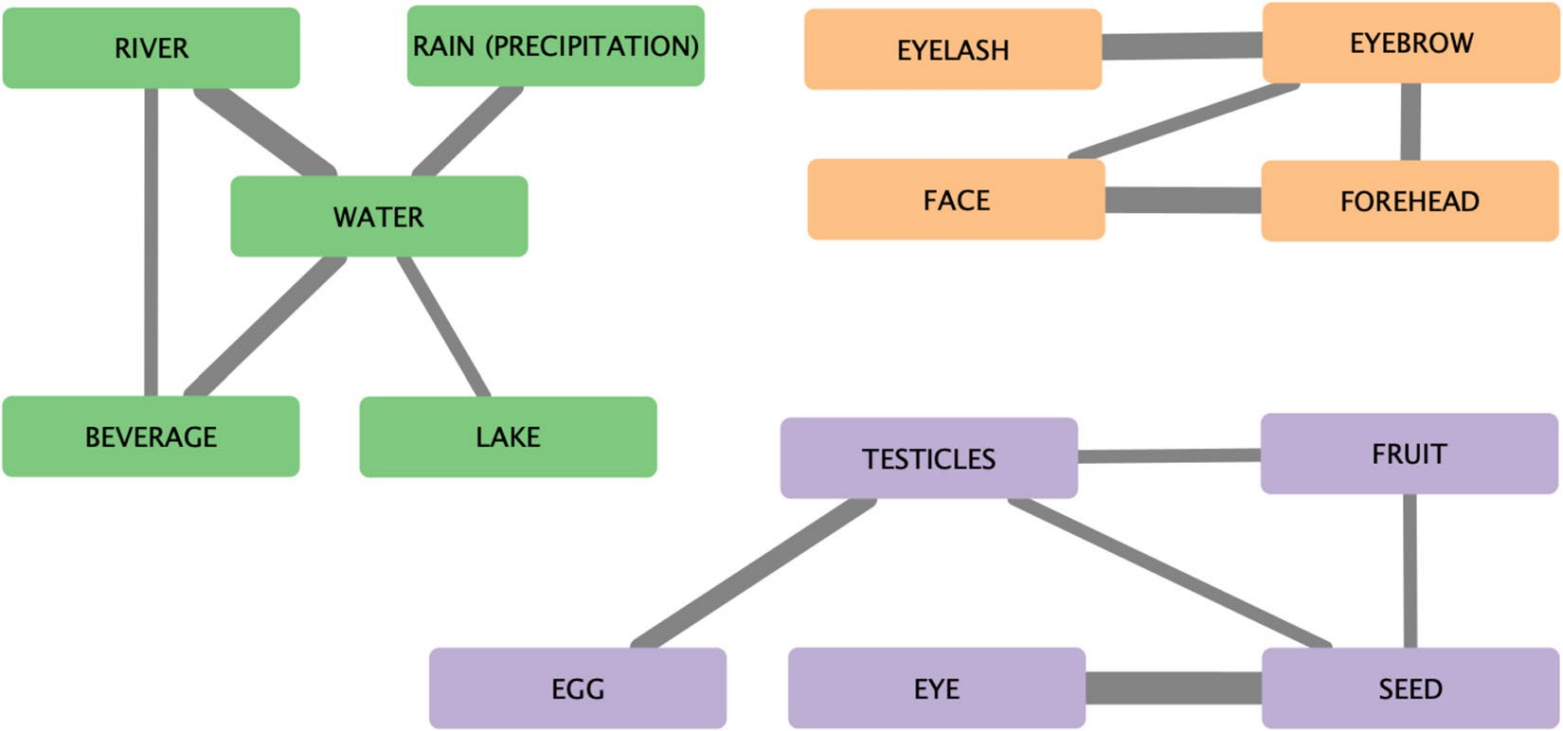
Colexification of concepts in the dataset. The width of the network edges corresponds to the amount of languages in which the concepts are colexified.

The standardization of the data makes it possible to study the colexifications in the dataset. A network visualization of those cross-semantic colexifications can be used to verify the semantic relationship between concepts in the language families of the dataset. In Fig. 3, we present the colexifications around the concepts WATER, EYE, and FACE.

## Usage Notes

Through the standardization of the data, we can exploit the consistency of the annotations in several ways. As the data is stored in tabular files (csv), the tables are readable on all computing platforms. You can install all the necessary packages that we use by cloning into the repository and installing the dependencies in the command line.

> git clone https://github.com/pano-tacanan-history/blumpanotacana

> cd blumpanotacana

> pip install –e.

The data can be accessed both manually and computationally. For a manual inspection of the data within a single file, we provide a ‘d_blumpanotacana.tsv‘ in the ‘analysis’-folder. This file includes the cognates and alignments, and can be uploaded to a local version of EDICTOR (‘http://lingulist.de/EDICTOR/’). This is especially useful for linguists who want to manually assess the quality of the alignments provided in the dataset. Of course, you can also open this file with any office application or use it for inspection with other tools of programming. The file is created using the ‘pyedictor’-package^61^ that comes as part of the repository dependencies, using the following command from the commandline interface:

> cd analysis/

> edictor wordlist --name = d_blumpanotacana --data = ./cldf/cldf-metadata.json > --preprocessing = s_realign.py --addon = “language_subgroup:subgroup”, > “cognacy:cogid”, “partial_cognacy:cogids”, “borrowing:borrowing”.

The call to pyedictor includes the output file (‘--name’), the input CLDF metadata (‘--data’), a script for pre-processing that can be adopted to other purposes (‘--preprocessing’), and columns from the different CLDF tables with the syntax ‘cldf-name:column-name’. The same workflow can also be used to create similar files from other Lexibank-datasets^8^.

Having installed the requirements, the dataset can now easily be converted to a SQLite dataset using a command from the pycldf-package in the command line^7^.

> cldf createdb cldf/cldf-metadata.json blumpanotacana.sqlite

This dataset can then be queried with all common programming and dataset tools. Given the linking to other reference catalogues in linguistics, the data is easily comparable with information from other datasets. For example, we can use SQLite queries to integrate the data with other datasets, such as Grambank^5^, the largest currently available dataset on grammatical information of languages, which is equally linked to the reference catalogues. By creating the SQLite dataset for Grambank in the same way as we did for the lexical dataset, we can retrieve the information in Grambank for all the languages in the dataset. This examplifies the utility advantage for integrating datasets by using CLDF and SQLite. The following SQLite commands showcases the integration of Grambank-data based on the glottocodes of the languages in the current dataset. An example script for this process that uses SQLite is provided within the ‘analysis/‘-folder.

> attach ‘blumpanotacana.sqlite’ AS db1;

> attach ‘grambank.sqlite’ AS db2;

> SELECT *

> FROM db1.LanguageTable AS a

> INNER JOIN db2.ValueTable AS b

> ON a.cldf_glottocode = b.cldf_languageReference;

## Data Availability

All code that has been used during the creation of this dataset is published on Zenodo (v0.2)^51^ and curated on GitHub (https://github.com/pano-tacanan-history/blumpanotacana). For converting the data to CLDF^7^, we have used the Python tools cldfbench (v1.13.0)^59^ using the pylexibank plugin (v3.4.0)^62^. The dataset is linked to Concepticon (v3.1.0)^40^, Glottolog (v4.7)^52^, and CLTS (v2.2.0)^53,57^. The code for integrating data with other datasets via SQL is presented in the main README.md. The scripts that were used to create the plots and to compute the coverage and synonymy is part of the ‘analysis’ folder, where another README.md file leads through the replication of all necessary steps. The code for the initial addition of IDS data is added to ‘raw/archive/‘ for documentation. This list was then filtered while finalizing the concept list. All the orthography profiles that are used during the conversion of graphemes are part of ‘etc/orthography’.
